# The Distribution of Match Physical Activities Relative to the Most Demanding Scenarios in Professional Basketball Players

**DOI:** 10.2478/hukin-2022-0059

**Published:** 2022-09-08

**Authors:** Franc García, Daniel Fernández, Jordi Illa, Xavier Reche, Jairo Vázquez-Guerrero

**Affiliations:** 1 Sports Performance Area, Futbol Club Barcelona, Barcelona, Spain; 2 Barça Innovation Hub, Futbol Club Barcelona, Barcelona, Spain

**Keywords:** team sport, competition, technology, external load

## Abstract

The purpose of this study was to examine the distribution of physical activities relative to the most demanding scenarios across playing positions during official basketball match-play. Twelve professional basketball players were monitored during twelve matches using a local positioning system. Peak physical demands were measured via total distance covered, distance covered >18 km·h^-1^, and the number of accelerations and decelerations >3 m·s^-2^ captured over 30- and 60-s rolling averages. The results showed that players were exposed to more than one high-demanding scenario in all variables and time epochs examined. Additionally, total distance covered presented the greatest number of moderate-demanding scenarios (40-80% of most demanding scenarios), whereas distance covered >18 km·h^-1^, and accelerations and decelerations >3 m·s^-2^ presented the greatest proportion of low-demanding scenarios (<40% of most demanding scenarios). Regarding positional differences, backcourt players generally experienced a higher number of scenarios than frontcourt players in most variables, especially in total distance covered. For this variable, scenarios between 20 and 70% of most demanding scenarios during the 30-s epoch (p < 0.001; ES = 0.420.78), and scenarios between 50 and 90% of most demanding scenarios during the 60-s epoch (p < 0.001; ES = 0.400.64) showed significant differences between backcourt and frontcourt players. Our data suggest that match physical activities are position-dependent and variable-dependent. In addition, peak physical demands appear to be repeated during basketball competition. These results may be considered by practitioners to complement average values and most demanding scenarios when prescribing individualized training programs to optimize team performance.

## Introduction

Basketball is an intermittent, dynamic, and complex court-based team sport that requires multidirectional explosive actions such as sprints, accelerations, jumps, and impacts. All these actions are based on specific movements, such as driving, lay-ups, jump shooting, fast breaking, closing out, and high-speed shot blocking (Gryko et al., 2020; Mikołajec et al., 2021; Ostojic et al., 2006). Additionally, previous research suggests that the playing position might influence physical demands during basketball competition, where frontcourt players tend to achieve greater workloads compared to backcourt players (Russell et al., 2020; Stojanović et al., 2018). Understanding the influence of position-specific physical demands during official matches is vital for sport medicine practitioners who aim to minimize the risk of injury and optimize sports performance (Soligard et al., 2016).

Previous studies reported that inappropriate workloads may lead to reduced team sport performance and an increased incidence of injuries (Caparrós et al., 2018; Gabbett, 2016). Therefore, a key task for strength and conditioning specialists, and for basketball coaches, is to periodize, design, implement, monitor, and manage training sessions, which ensures that players will be prepared to deal with match intensities. Training prescription is broadly focused on the match activity profile, commonly derived from the traditional average demands during competition. Owing to the use of inertial devices combined with ultra-wide band-based local positioning systems to track players indoor (Serpiello et al., 2017), average values of distance (based on distance covered at different speed zones), and the number of high-intensity accelerations and decelerations during basketball match play have been extensively examined (García et al., 2020; Svilar et al., 2018; Vázquez-Guerrero et al., 2019).

The traditional approach of average physical demands was demonstrated to underestimate peak intensities during basketball match play (Gabbett et al., 2016; Vázquez-Guerrero & Garcia, 2020); therefore, it seems necessary to use alternative methods to determine the most demanding scenarios (MDS) and to prescribe appropriate training intensities during basketball-specific drills (Vázquez-Guerrero et al., 2020). With the advancement of micro-technology, there has been a large growth in research using rolling averages or moving averages (Fox et al., 2020a), to examine peak competition demands of team sports, based on the identification of the MDS (Vázquez-Guerrero et al., 2020), also referred as the most demanding passages in the current literature (Fernández et al., 2020; Illa et al., 2020b). This novel approach provides vital information that may complement previous research mostly focused on traditional average physical demands of basketball match play.

Rolling average techniques have frequently been used to determine the peak demands during different time epochs, such as at 30 and 60 s, in both outdoor (Johnston et al., 2020; Thornton et al., 2020; Whitehead et al., 2018) and indoor sports, such as basketball (Alonso et al., 2020; Fox et al., 2020a, 2020b; Vázquez-Guerrero et al., 2020), rink hockey (Fernández et al., 2020), and futsal (Illa et al., 2020a). Most of the investigations which reported peak demands sought to determine only one maximal scenario per game, obviating that a greater number of scenarios of the same (or similar) magnitude were also possible and meaningful. This circumstance occurs when players are exposed to more than one scenario which is remarkably close to the MDS. This concept has been previously described as the repetition of high and very high demanding scenarios (80-90% and >90% of the MDS) (Illa et al., 2020a). To the authors’ knowledge, only two investigations have reported the distribution of submaximal intensities relative to the peak game demands in Australian football and rugby league (Johnston et al., 2020; Thornton et al., 2020). Nevertheless, to date, no study has described the distribution of the physical activities relative to the peak demands during basketball competition.

Therefore, the aim of this study was to determine the 30- and 60-s MDS, the distribution of distance, the distance covered >18 km·h^-1^, and the number of accelerations and decelerations > 3 m·s^-2^, relative to the maximal mean intensities, in frontcourt and backcourt players in professional basketball during official competition.

## Methods

### Participants

Twelve professional male basketball players (age: 19.59 ± 1.65 years; body mass: 93.42 ± 15.45 kg; body height: 200.86 ± 7.87 cm; all measurements: mean ± SD) from a team which competed in the Spanish basketball second league voluntarily participated in the study. Following Russell et al.’s (2020) recommendations, players were categorized according to their playing position as frontcourt (n = 7) and backcourt (n = 5) players. For data to be included in our analysis, players had to participate for a minimum of five minutes of live time, not suffering injury during the match (Vázquez-Guerrero et al., 2019, 2020; Vázquez-Guerrero and García, 2020). These players were routinely monitored as part of their day-to-day training and playing practices. Prior to the commencement of the study, all participants were fully informed of the purpose and requirements of the study and they provided voluntary written informed consent before participating. Therefore, no authorization was required form an institutional ethics committee (Winter and Maughan, 2009), and this study was conducted in accordance with the Declaration of Helsinki.

### Design and Procedures

The current retrospective observational study was designed to profile the distribution of match physical activities of male professional basketball players, in relation to the MDS of competitive match play. Data from 12 official LEB Oro matches (Spanish second basketball league) were collected through an electronic performance and tracking system (WIMU PRO™, Realtrack Systems, Almeria, Spain) during the 2018-2019 competitive season. Specifically, the team won 2 out of 12 home games analyzed and finished the regular season in the 17^th^ position after winning 9 out of the 34 total matches.

### Measures

**Figure 1:**
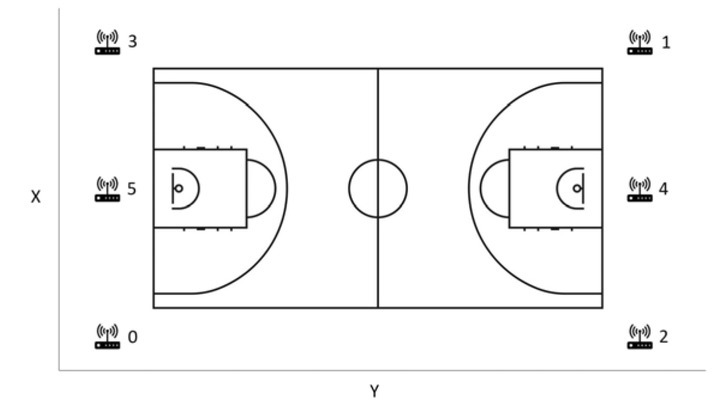
Ultra-wide band positioning system on a basketball court. Note: X is court width, y is court length and z is height of the antenna. Numbers show the disposition of antennas in cm: 0 is x = 0, y = 0, z = 600; 1 is x = 2924, y = 5208, z = 600; 2 is x = 0, y = 5208, z = 600; 3 is x = 2928, y = 7, z = 600; 4 is x = 1469, y = 5207, z = 600; and 5 is x = 1456, y = 2, z = 600.

Players' physical demands were monitored and recorded with a local positioning system (LPS) (WIMU PRO™, Realtrack Systems SL) during twelve competitive home matches. Based on recent recommendations regarding data collection quality (Rico-González et al., 2020), this research observed certain considerations: (1) the technology used was ultra-wide band (UWB), which occupied a very large frequency band, at least 0.5 GHz; (2) for analysis, the UWB system was calibrated, and the WIMU PRO devices were synchronized to the UWB system one hour before the game started, through the antennas and the technology; (3) there was no metallic material in the vicinity of the antennas; and (4) the system used time difference of arrival (TDOA), one of the most widely-used localization schemes that records the arrival time of the source signal. Throughout the course of the season, each player wore the same assigned LPS micro-technology device (81 x 45 x 15 mm, 70 g) to reduce any potential inter-unit variability (Castellano et al., 2011). The WIMU PRO™ units were equipped with four 3D accelerometers (full-scale out output ranges were ± 16 g, ± 16 g, ± 32 g, ± 400 g; 100 Hz sample frequency), three gyroscopes (8000º/s full-scale output range; 100 Hz sample frequency), a 3D magnetometer (100 Hz sample frequency), a global positioning system (10 Hz sample frequency), and a LPS with UWB technology (18 Hz sample frequency). For better signal emission and reception, the LPS installed on the basketball court consisted of six UWB antennas, which were located forming a rectangle (Figure 1) (Vázquez-Guerrero and García, 2020). With a sampling frequency for positioning data of 18 Hz, the LPS operated using triangulations between the antennas and the units (the six antennas sent a signal to the units every 55.5 ms). Then, the device calculated the time required to receive the signal and derived the unit position (coordinates X and Y) using one of the antennas as a reference.

All matches were played on the same court with similar environmental conditions. The LPS units were activated ten minutes prior to the start of each match, after a standardized 30-min warm-up. These units were then fitted to players within a specially designed tight-fit vest between the shoulder blades in the upper part of the back, ensuring that players' torso and upper limb mobility was not restricted. All players were continuously monitored during each match; however, real-time LPS data were only considered for analysis when players were competing on the court, excluding the resting periods between quarters and every time players were substituted.

The four physical demand variables selected to describe the distribution of match activities were: total distance covered (m), distance covered >18 km·h^-1^ (m), and the number of accelerations and decelerations >3 m·s^-2^.

After each match, LPS data were downloaded and extracted using manufacturer's software (SPRO™, Realtrack Systems SL, version 956). Software provided instantaneous raw data for each variable and player, using the rolling average method with two different time epochs (30 s and 60 s), and recording from the greatest to the smallest time epoch values. Rolling averages are used in a variety of team sports (Fernández et al., 2020; Illa et al., 2020a; Vázquez-Guerrero et al., 2020; Whitehead et al., 2018) to calculate most demanding scenarios during a pre-fixed time epoch. For example, for a 60 s epoch, WIMU PRO™ software identified 1080 consecutive data points (e.g., 18 samples/s for 60 s) to calculate the player’s greatest relative demand. In each epoch length, the peak values of physical demands selected were recorded independently, hence it was very likely that they came from different data points. The 30-s epoch was chosen as it represents the average duration of continuous playing in professional basketball, even though longer scenarios up to 120 s are infrequent, but possible (Salazar and Castellano Paulis, 2020). In addition to the fact that the 60-s epoch was already used in previous research (Fox et al., 2020a; Vázquez-Guerrero and García, 2020), its choice is also justified by the possibility to compare the data obtained in this epoch with common average physical demands during competition.

Based on the research of Illa et al. (2020a, 2020b), the data computation procedures were structured in two steps, which were always applied for each player and each variable. The first step was to determine an individual reference value (100% most demanding scenario reference value). For this purpose, a mean of the top three observations was examined, to smooth possible outliers (the individual reference value would have been distorted if only the greatest scenario of all games had been considered), but not to smooth the individual reference value too much (the value would have been excessively smoothed if all the observations of all games had been included into the mean). The results of these 100% reference values are presented in Tables 1 and 2 in comparison with the mean of the most demanding scenarios from all games, the maximum most demanding scenario, and the minimum most demanding scenario. The second step was to establish 10% buckets based on the individual 100% reference values and count the number of scenarios on each bucket. The final output was the total number of scenarios for each bucket, player, and variable.

**Table 1:** Individual reference values for total distance covered and distance covered >18 km·h^-1^.

			Total Distance covered (m)				Distance covered > 18 km ·h^-1^ (m)			
			
T. epoch	Position	Player	Top 3	All	Max	Min	Top 3	All	Max	Min
	Backcourt	P1	89.76	86.54	90.83	78.17	21.96	18.84	26.66	12.87
	Backcourt	P2	90.54	83.87	91.88	75.18	25.05	18.54	26.70	13.15
	Backcourt	P3	99.46	87.72	103.70	75.54	38.46	25.88	41.85	18.00
	Backcourt	P4	101.03	93.43	116.38	69.13	35.64	26.41	38.86	20.17
	Backcourt	P5	103.86	91.77	115.11	78.37	33.11	23.91	35.42	14.42
	Backcourt	P6	91.09	82.51	94.76	75.05	21.63	16.72	23.57	12.39
	Backcourt	P7	87.38	78.24	93.57	67.09	21.40	16.23	23.80	11.19
		
		Mean	94.73	86.30	100.89	74.08	28.18	20.93	30.98	14.60
		SD	6.04	4.90	10.16	4.01	6.79	4.02	7.01	3.03
30-s										
	Frontcourt	P8	81.67	81.67	82.05	81.28	26.83	26.38	37.54	14.66
	Frontcourt	P9	87.45	81.27	83.80	65.07	28.31	19.95	31.75	13.98
	Frontcourt	P10	79.31	73.13	96.36	78.36	26.32	19.14	27.36	9.67
	Frontcourt	P11	91.07	84.65	89.22	67.10	24.28	18.66	24.96	9.05
	Frontcourt	P12	86.56	77.73	93.57	67.09	25.53	18.56	27.17	12.30
		
		Mean	85.21	79.69	89.00	71.78	26.25	20.54	29.76	11.93
		SD	4.70	4.42	6.13	7.45	1.50	3.31	5.00	2.51

										
	Backcourt	P1	153.86	147.51	157.60	133.19	23.68	20.27	26.66	14.92
	Backcourt	P2	146.18	140.08	149.17	132.86	30.02	21.85	31.37	14.40
	Backcourt	P3	158.51	147.09	160.31	134.47	38.77	28.70	41.85	18.00
	Backcourt	P4	158.27	148.27	171.03	120.90	38.96	29.18	40.02	20.20
	Backcourt	P5	168.50	154.78	180.16	141.53	36.17	28.25	39.50	17.61
	Backcourt	P6	146.49	135.85	154.34	128.73	24.22	19.06	26.09	12.39
	Backcourt	P7	143.16	131.96	149.68	114.30	27.23	20.68	30.18	11.19
		
		Mean	153.57	143.65	160.33	129.43	31.29	24.00	33.67	15.53
		SD	8.31	7.39	10.62	8.45	6.15	4.16	6.16	2.99
60-s										
	Frontcourt	P8	134.46	133.32	141.25	126.13	27.45	27.08	37.54	16.75
	Frontcourt	P9	148.06	135.48	149.70	124.78	30.40	22.94	32.74	14.92
	Frontcourt	P10	134.07	122.50	139.35	112.77	26.47	20.06	27.36	11.70
	Frontcourt	P11	152.91	141.86	160.17	131.82	30.74	23.23	35.09	13.31
	Frontcourt	P12	144.31	132.48	145.68	115.87	31.47	23.63	32.28	15.52
		
		Mean	142.76	133.13	147.23	122.27	29.31	23.39	33.00	14.44
		SD	8.34	6.99	8.27	7.80	2.20	2.50	3.79	1.97

*Note: Mean of the three most demanding scenarios for each player (top3); mean of all mostdemanding scenarios for each player (all); the maximum registered scenario; and the minimum registered scenario; Values reported by time epoch and position*

**Table 2:** Individual reference values for accelerations and decelerations >3 m·s^-2^.

			Accelerations >3 m·s^-2^ (n)				Decelerations >3 m·s^-2^ (n)			
			
T. epoch	Position	Player	Top 3	All	Max	Min	Top 3	All	Max	Min
	Backcourt	P1	3.7	3.5	4.0	3.0	3.3	3.3	4.0	3.0
	Backcourt	P2	4.0	3.5	4.0	3.0	4.3	3.8	5.0	3.0
	Backcourt	P3	5.3	3.5	6.0	2.0	4.3	3.6	5.0	3.0
	Backcourt	P4	5.7	4.3	6.0	3.0	5.7	4.7	7.0	2.0
	Backcourt	P5	6.0	4.1	7.0	3.0	6.3	4.5	7.0	3.0
	Backcourt	P6	4.7	3.4	5.0	1.0	4.0	3.5	4.0	2.0
	Backcourt	P7	5.0	3.5	5.0	3.0	5.0	3.9	5.0	3.0
		
		Mean	4.90	3.70	5.29	2.57	4.71	3.89	5.29	2.71
		SD	0.79	0.34	1.03	0.73	0.95	0.48	1.16	0.45
30-s	Frontcourt									
		P8	5.0	4.0	5.0	2.0	4.3	3.3	5.0	1.0
	Frontcourt	P9	4.7	3.4	5.0	2.0	4.3	3.4	5.0	2.0
	Frontcourt	P10	5.0	3.4	5.0	1.0	4.0	3.3	4.0	1.0
	Frontcourt	P11	4.3	3.0	5.0	2.0	3.7	3.0	4.0	2.0
	Frontcourt	P12	5.0	3.8	5.0	3.0	4.7	3.8	5.0	3.0
		
		Mean	4.80	3.51	5.00	2.00	4.20	3.36	4.60	1.80
		SD	0.30	0.40	0.00	0.71	0.38	0.30	0.55	0.84

	Backcourt	P1	5.0	4.8	6.0	4.0	4.0	3.8	5.0	3.0
	Backcourt	P2	5.3	4.5	6.0	3.0	5.3	4.9	6.0	4.0
	Backcourt	P3	7.0	4.5	8.0	3.0	6.0	4.2	6.0	3.0
	Backcourt	P4	7.3	6.0	8.0	4.0	8.3	5.7	9.0	3.0
	Backcourt	P5	9.0	5.8	11.0	4.0	8.3	6.0	9.0	4.0
	Backcourt	P6	5.7	4.2	6.0	2.0	4.7	4.1	5.0	3.0
	Backcourt	P7	7.7	4.8	8.0	3.0	7.7	5.2	8.0	3.0
		
		Mean	6.71	4.93	7.57	3.29	6.33	4.82	6.86	3.29
		SD	1.34	0.64	1.68	0.70	1.65	0.79	1.64	0.45
60-s	Frontcourt	P8	6.7	4.7	7.0	2.0	6.7	5.0	8.0	1.0
	Frontcourt	P9	6.3	4.1	7.0	2.0	5.3	3.7	6.0	2.0
	Frontcourt	P10	6.7	4.5	8.0	1.0	6.3	4.8	7.0	1.0
	Frontcourt	P11	6.0	4.0	7.0	2.0	5.0	3.8	6.0	2.0
	Frontcourt	P12	7.3	5.3	8.0	4.0	6.7	4.7	8.0	4.0
		
		Mean	6.60	4.50	7.40	2.20	6.00	4.39	7.00	2.00
		SD	0.49	0.50	0.55	1.10	0.78	0.58	1.00	1.22

*Note: Mean of the three most demanding scenarios for each player (top3); mean of all most demanding scenarios for each player (all); the maximum registered scenario; and the minimum registered scenario; Values reported by time epoch and position*

### Statistical Analysis

All statistical analyses were conducted with RStudio version 1.3.1093 (RStudio, Inc.). Descriptive data were reported as mean ± standard deviation. To perform the hypothesis test to assess the differences between positions, a bootstrap confidence interval approach was used (Jovanović, 2020; Wilcox, 2010). A resampling model, with 2,000 bootstrap samples and the 95% bias-corrected and accelerated method, was used to calculate the confidence intervals of *t*-test values for each variable; the null hypothesis was that there were no differences between positions; and the mean difference in the number of scenarios was computed and presented as standardized differences (Cohen’s d). Thresholds for standardized differences statistics were <0.20, trivial; 0.20–0.59, small; 0.60–1.19, moderate; 1.20– 1.99, large; and >2.0, very large (Hopkins et al., 2009). All the reported *p*-values represent the likelihoods to observe the absolute effect sizes if the null hypothesis of zero difference was true (Plonsky, 2015).

## Results

**Figure 2:**
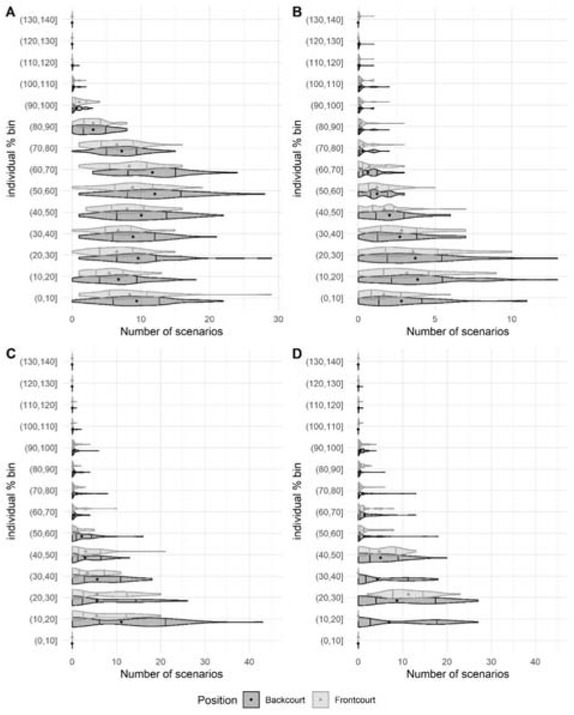
Distribution (violin plot), quartiles 25 and 75 (vertical lines), and mean (point or triangle, depending on the player’s position) of the number of scenarios for each bucket, position, and variable, in the 30-s epoch. Note = (A) total distance covered, (B) distance covered >18 km·h^-1^, (C) accelerations >3 m·s^-2^, (D) decelerations >3 m·s^-2^.

**Figure 3:**
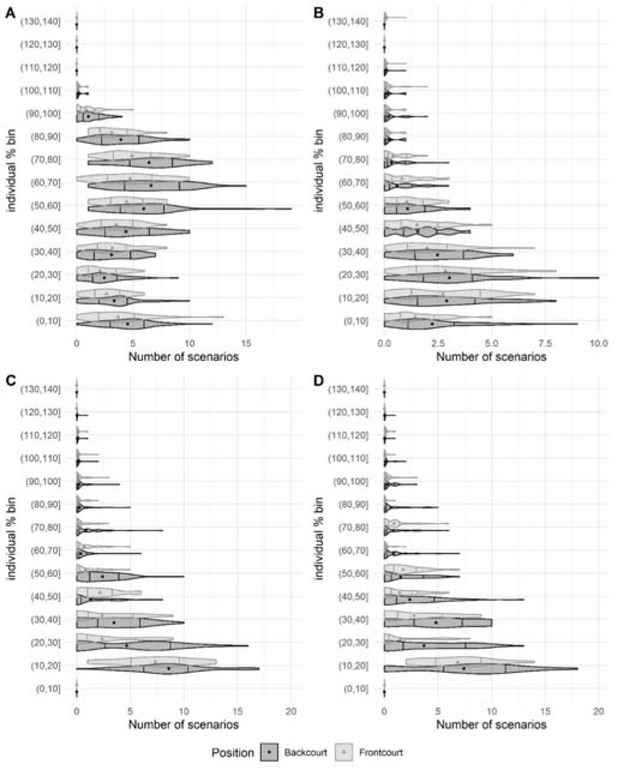
Distribution (violin plot), quartiles 25 and 75 (vertical lines), and mean (point or triangle, depending on the player’s position) of the number of scenarios for each bucket, position, and variable, in the 60-s epoch. Note = (A) total distance covered, (B) distance covered >18 km·h^-1^, (C) accelerations >3 m·s^-2^, (D) decelerations >3 m·s^-2^.

The descriptive data of the number of scenarios in each bucket and time epoch for each variable are presented in Tables 3 and 4, and Figures 2 and 3. In the two time epochs analysed, the total distance covered variable was the metric with the highest number of scenarios in the central area of the buckets (between 50–60% and 60–70%), and distance covered >18 km·h^-1^. Accelerations and decelerations >3 m·s^-2^ presented the greatest number of scenarios between 0 and 40% buckets.

**Table 3:** Number (mean ± standard deviation) of scenarios for each time epoch and bucket, for total distance covered and distance covered >18 km·h^-1^.

T. epoch	Bucket	Total Distance Covered (m)		Distance Covered > 18 km ·h^-1^ (m)	
		Frontcourt	Backcourt	Frontcourt	Backcourt
		
	(0,10]	8.36 ± 4.90	9.39 ± 4.80	1.66 ± 1.41	2.82 ± 2.54*
	(10,20]	5.39 ± 2.95	6.74 ± 3.97*	3.16 ± 2.26	3.87 ± 2.77
	(20,30]	6.48 ± 3.29	9.61 ± 4.98*	3.57 ± 2.52	3.72 ± 2.73
	(30,40]	6.68 ± 3.24	8.87 ± 4.23*	2.82 ± 1.86	2.70 ± 1.90
	(40,50]	7.98 ± 3.56	10.07 ± 4.89*	1.68 ± 1.52	2.03 ± 1.57
	(50,60]	8.77 ± 4.18	12.03 ± 5.67*	1.20 ± 1.09	1.23 ± 0.88
30-s	(60,70]	8.30 ± 3.91	11.69 ± 4.65*	0.70 ± 1.00	0.64 ± 0.78
	(70,80]	6.50 ± 3.31	7.23 ± 3.18	0.32 ± 0.67	0.28 ± 0.49
	(80,90]	3.05 ± 1.89	3.08 ± 2.25	0.20 ± 0.59	0.15 ± 0.40
	(90,100]	1.02 ± 1.19	0.61 ± 0.80*	0.11 ± 0.39	0.10 ± 0.30
	(100,110]	0.16 ± 0.43	0.13 ± 0.39	0.16 ± 0.37	0.15 ± 0.40
	(110,120]	0.00 ± 0.00	0.03 ± 0.18	0.02 ± 0.15	0.02 ± 0.13
	(120,130]	0.00 ± 0.00	0.00 ± 0.00	0.00 ± 0.00	0.02 ± 0.13
	(130,140]	0.00 ± 0.00	0.00 ± 0.00	0.02 ± 0.15	0.00 ± 0.00

	(0,10]	3.66 ± 2.59	4.52 ±2.25	1.43 ±1.28	2.23 ±1.88*
	(10,20]	2.64 ± 1.63	3.33 ±2.22	2.73 ±2.00	2.90 ±1.95
	(20,30]	2.07 ± 1.65	2.44 ± 1.83	2.84 ± 2.00	3.03 ± 2.01
	(30,40]	3.14 ± 2.01	3.08 ± 2.04	1.98 ± 1.44	2.48 ± 1.60
	(40,50]	3.50 ± 1.85	4.36 ± 2.79	1.52 ± 1.34	1.56 ± 1.22
	(50,60]	4.43 ± 2.08	5.95 ±3.35*	1.05 ± 0.96	1.08 ±1.10
	(60,70]	4.73 ± 2.68	6.61 ± 3.07*	0.82 ± 0.95	0.59 ± 0.82
60-s	(70,80]	4.89 ± 2.27	6.41 ±2.89*	0.41 ± 0.62	0.33 ± 0.63
	(80,90]	3.09 ± 1.74	3.92 ± 2.27*	0.14 ± 0.35	0.20 ± 0.40
	(90,100]	0.82 ± 1.04	1.05 ± 1.02	0.14 ± 0.35	0.20 ± 0.48
	(100,110]	0.14 ± 0.35	0.15 ± 0.36	0.11 ± 0.39	0.13 ± 0.34
	(110,120]	0.00 ± 0.00	0.00 ± 0.00	0.02 ± 0.15	0.03 ± 0.18
	(120,130]	0.00 ± 0.00	0.00 ± 0.00	0.00 ± 0.00	0.00 ± 0.00
	(130,140]	0.00 ± 0.00	0.00 ± 0.00	0.02 ± 0.15	0.00 ± 0.00

Note: significant bootstrap t-test comparisons (p < 0.05) are bolded and marked with a *

**Table 4:** Number (mean ± standard deviation) of scenarios for each time epoch and bucket, for accelerations and decelerations >3 m·s^-2^.

T. epoch	Bucket	Accelerations > 3 m·s^-2^ (n)		Decelerations > 3 m·s^-2^ (n)	
		Frontcourt	Backcourt	Frontcourt	Backcourt
		
	(0,10]	0.00 ± 0.00	0.00 ± 0.00	0.00 ± 0.00	0.00 ± 0.00
	(10,20]	5.55 ± 6.43	11.11 ± 10.62*	0.00 ± 0.00	6.93 ± 9.03*
	(20,30]	5.59 ± 6.50	5.62 ± 7.71	11.30 ± 4.50	8.74 ± 8.70*
	(30,40]	3.43 ± 3.84	5.67 ± 5.68*	0.00 ± 0.00	4.30 ± 5.92*
	(40,50]	3.02 ± 4.41	2.92 ± 3.51	4.73 ± 4.03	5.05 ± 4.74
	(50,60]	1.14 ± 1.61	2.20 ± 3.41*	1.18 ± 2.41	1.46 ± 3.32
30-s	(60,70]	0.89 ± 1.85	0.54 ± 1.12	1.32 ± 1.99	1.43 ± 2.31
	(70,80]	0.43 ± 0.76	1.02 ± 1.92*	0.43 ± 1.21	1.02 ± 2.35
	(80,90]	0.16 ± 0.48	0.36 ± 0.86	0.50 ± 0.76	0.16 ± 0.82*
	(90,100]	0.34 ± 0.86	0.28 ± 0.90	0.45 ± 0.95	0.51 ± 0.79
	(100,110]	0.05 ± 0.21	0.10 ± 0.35	0.09 ± 0.29	0.00 ± 0.00*
	(110,120]	0.02 ± 0.15	0.03 ± 0.18	0.05 ± 0.21	0.07 ± 0.25
	(120,130]	0.00 ± 0.00	0.00 ± 0.00	0.00 ± 0.00	0.02 ± 0.13
	(130,140]	0.00 ± 0.00	0.00 ± 0.00	0.00 ± 0.00	0.00 ± 0.00

	(0,10]	0.00 ± 0.00	0.00 ± 0.00	0.00 ± 0.00	0.00 ± 0.00
	(10,20]	7.32 ± 3.30	8.61 ± 3.59	6.84 ± 3.00	7.41 ± 4.97
	(20,30]	2.36 ± 3.00	4.67 ± 4.41*	1.50 ± 2.52	3.69 ± 3.70*
	(30,40]	2.36 ± 2.87	3.49 ± 2.76*	2.77 ± 2.98	4.80 ± 3.49*
	(40,50]	2.16 ± 1.87	1.31 ± 2.13*	1.41 ± 1.83	2.36 ± 2.74*
	(50,60]	0.82 ± 1.26	2.41 ± 2.05*	1.73 ± 1.68	1.51 ± 1.95
	(60,70]	0.70 ± 1.13	0.41 ± 1.15	0.11 ± 0.39	0.85 ± 1.55*
60-s	(70,80]	0.32 ± 0.71	0.85 ± 1.44*	0.89 ± 1.26	0.82 ± 1.42
	(80,90]	0.18 ± 0.45	0.28 ± 0.84	0.05 ± 0.21	0.64 ± 1.27*
	(90,100]	0.18 ± 0.54	0.23 ± 0.69	0.25 ± 0.72	0.31 ± 0.65
	(100,110]	0.07 ± 0.33	0.08 ± 0.38	0.02 ± 0.15	0.10 ± 0.35
	(110,120]	0.07 ± 0.25	0.05 ± 0.22	0.11 ± 0.32	0.02 ± 0.13
	(120,130]	0.00 ± 0.00	0.02 ± 0.13	0.00 ± 0.00	0.02 ± 0.13
	(130,140]	0.00 ± 0.00	0.00 ± 0.00	0.00 ± 0.00	0.00 ± 0.00

Note: significant bootstrap t-test comparisons (p < 0.05) are bolded and marked with a *

**Figure 4:**
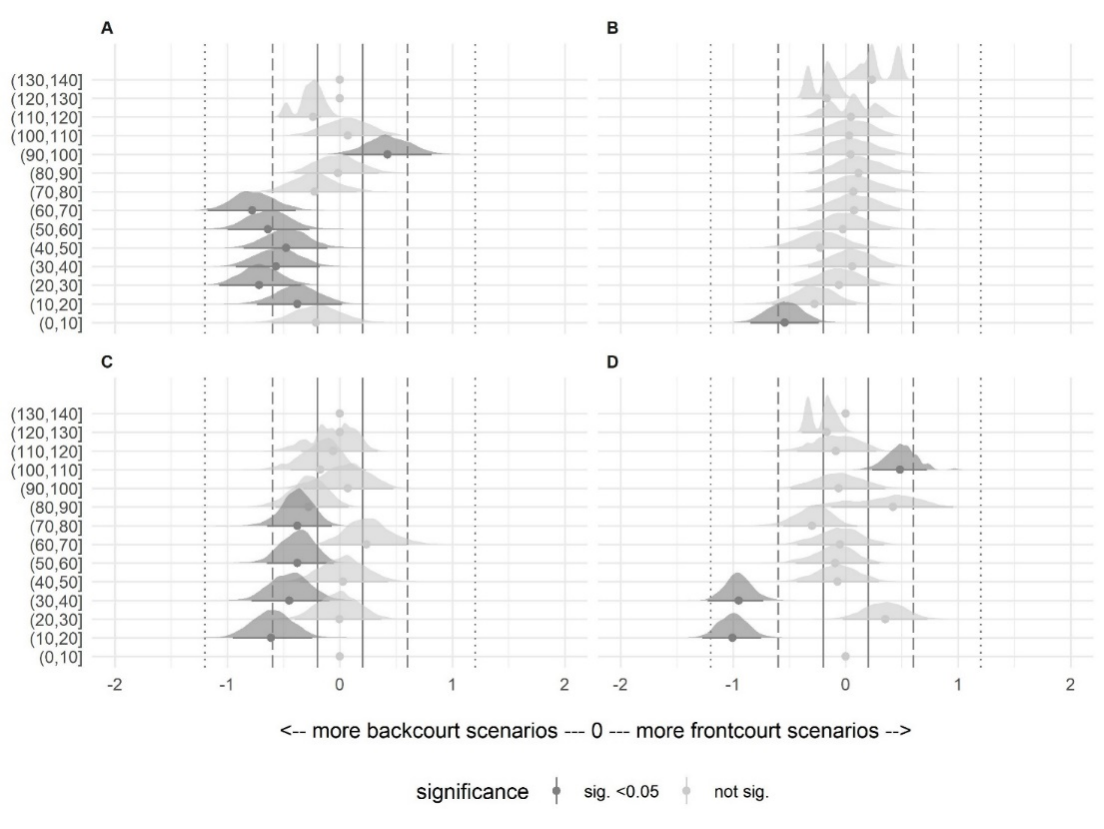
Standardized differences (Cohen’s d), and the 95% confidence interval between positions, in all analyzed buckets in the 30-s epoch. Grey lines include trivial effects, dashed lines include small effects, and dotted lines include moderate effects. Note = (A) total distance covered, (B) distance covered >18 km·h^-1^, (C) accelerations >3 m·s^-2^, (D) decelerations >3 m·s^-2^.

**Figure 5:**
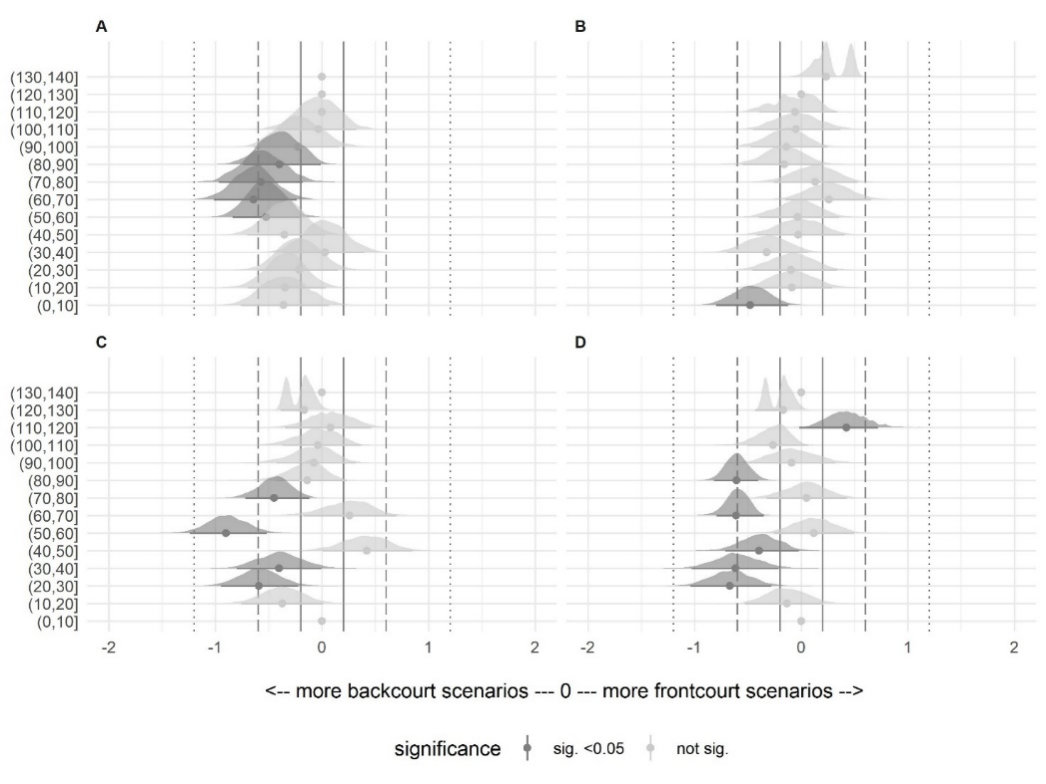
Standardized differences (Cohen’s d), and the 95% confidence interval between positions, in all analyzed buckets in the 60-s epoch. Grey lines include trivial effects, dashed lines include small effects, and dotted lines include moderate effects. Note = (A) total distance covered, (B) distance covered >18 km·h^-1^, (C) accelerations >3 m·s^-2^, (D) decelerations >3 m·s^-2^.

Standardized differences (Cohen’s d) and 95% confidence intervals between playing positions for all the variables and buckets are presented in Figures 4 and 5 for the 30- and 60-s epochs, respectively. In all buckets with significant differences, backcourt players had more scenarios with standardized effects thresholds between moderate and small, than frontcourt players. Exceptions were represented by the total distance covered in the 30-s epoch in the 90–100% bucket, and the number of decelerations >3 m·s^-2^ in 30- and 60-s epochs in the 110–120% bucket.

## Discussion

The aim of this study was to determine the distribution of match physical demands relative to the most demanding scenarios, across playing positions in 30- and 60-s epochs in professional basketball. The primary finding of this study is that, during professional basketball competition, peak physical demands did not occur as an isolated situation during the match. Indeed, players were exposed to more than one high or very high-demanding scenario during a single match. Additionally, our data reveal that the distribution of the match physical activities relative to the MDS is variable-dependent during 30- and 60-s epochs, with a common tendency to decrease in the number of scenarios as the threshold approaches 100% of the MDS. Besides, multiple significant differences were found between frontcourt and backcourt players. Understanding the distribution of match physical activities relative to the MDS across playing positions might be useful to optimize the individual players’ and team performance.

The consideration of the MDS during competition can complement the traditional approach based on average values to design optimal training drills (Vázquez-Guerrero et al., 2020; Vázquez-Guerrero and García, 2020); moreover, previous research conducted in indoor team-sports (Illa et al., 2020a, 2020b) concluded that high and very demanding scenarios repeatedly occurred during professional futsal matches. Along with the findings of Illa et al. (2020a), the results of our study show a similar trend in terms of repeatability of scenarios for total distance covered. Indeed, total distance covered presented higher repeatability of high-demanding scenarios compared to high-intensity variables, such as distance covered >18 km·h**^-1^**, and acceleration and deceleration actions >3 m·s**^-2^**. Although these results could be attributed to the basketball idiosyncrasy and the team’s specific playing model, which is characterized by fast-paced game play, the fact that futsal (Illa et al., 2020a, 2020b) presented comparable results could add a justification in relation to the high-volume and low-intensity nature of the locomotor total distance. More specifically, total distance covered presented up to three high-demanding scenarios during 30- and 60-s epochs. On the contrary, distance covered >18 km·h**^-1^**, and accelerations and decelerations >3 m·s**^-2^** showed lower repeatability of high-demanding scenarios, with less than one scenario >80% of the MDS across playing positions and time epochs.

Regarding the match physical activity profile, total distance covered showed the greatest proportion of scenarios between 40 and 80% of the MDS. On the contrary, distance covered >18 km·h**^- 1^**, and accelerations and decelerations >3 m·s**^-2^** presented most activities at <40% of the MDS. Johnston et al. (2020) found the greatest volume of total distance covered at 60% of the MDS, in a 60-s epoch in the rugby league and Australian football. In agreement with Johnston et al.’s (2020) findings, our results reported that total distance covered followed a normal distribution, with the highest repeatability in moderate-demanding scenarios (40-80% of the MDS) and decreasing thereafter. Conversely, most of the scenarios in distance covered >18 km·h**^-1^**, and accelerations and decelerations >3 m·s**^-2^**, were considered low-demanding (0-40% of the MDS). These observations show the importance of variable selection to adequately represent the components of the load’s nature (e.g., volume and intensity), and to better understand the total distribution of physical demand activities relative to the MDS of match-play.

When comparing the distribution of the MDS across physical demands, time epochs, and playing positions, there were multiple significant differences between backcourt and frontcourt players (Figures 2 and 3). Similar to previous research in basketball (Stojanović et al., 2018), our data revealed that backcourt players tended to be exposed to higher physical demands than frontcourt players, possibly due to a combination of anthropometric, technical, and tactical characteristics, according to the playing model. While backcourt players are usually smaller and lighter than frontcourt players (Russell et al., 2020), they must perform a greater number of continuous high-intensity movements, such as full-court defense, one-on-one attacks, and actions after different types of screens (Sampaio et al., 2006). On the other hand, frontcourt players present higher values in distance covered >18 km·h**^-1^**, jumps and impacts than backcourt players (García et al., 2020). In addition to their typical role of setting screens, rebounding and shot-blocking, frontcourt players have adapted a more versatile play-style where they are required to perform switch defense, play in the perimeter and cover more distance at high-speed intensities in offensive and defensive transitions. Regarding differences, the four variables (total distance covered, distance covered >18 km·h**^-1^**, and accelerations and decelerations >3 m·s**^-2^**) presented a minimum of one significant difference with small to moderate effect size between playing positions across the 10% buckets used. Specifically, most differences were found in total distance covered: backcourt players achieved significantly lower and more moderate (20-70% of the MDS) demanding scenarios during the 30-s epoch (p < 0.001; ES = 0.42-0.78, small to moderate difference); and moderate to high (50-90% of the MDS) demanding scenarios during the 60-s epoch (p < 0.001; ES = 0.40-0.64, small to moderate difference), compared to frontcourt players. To the best of authors’ knowledge, this is the first study in basketball which has included playing positions to examine how match physical activities are distributed in relation to the most demanding scenarios. Our results show that the distribution of physical activities seems to be position-dependent in professional basketball players during official competition.

In conclusion, this study shows that basketball matches tend to include more than one exposure to high and very high-demanding scenarios which are closed to the peak physical demands of match-play. Additionally, our data reveal different distributions of match physical activities relative to the MDS, during the 30- and 60-s epochs across playing positions. Different distributions were detected between total distance covered, distance covered >18 km·h**^-1^**, and accelerations and decelerations >3 m·s**^-2^**. In addition to the MDS, strength and conditioning professionals and basketball coaches must consider multiple high-demanding scenarios during competition, and variability of match-play activities relative to the peak physical demands, when optimizing training and match performance. Specifically, up to three situations >80% of the MDS of match-play in total distance covered, and a minimum of one situation of similar intensity in distance covered >18 km·h**^-1^**, and in the number of accelerations and decelerations >3 m·s**^-2^**, should appear during training in basketball-specific 30- and 60-s epochs. Furthermore, caution should be taken when prescribing skill-based basketball drills aiming to simulate the distribution of different variables relative to the MDS of match-play. In this regard, total distance covered demands higher attention to moderate-intensity scenarios (40-80% of the MDS), whereas distance covered >18 km·h**^-1^**, and decelerations >3 m·s**^-2^** demand higher attention to low-demanding scenarios (<40% of the MDS). Finally, when prescribing individualized training interventions based on specific playing positions, it seems vital to protect frontcourt players and do not force them to accumulate similar physical loads to backfront players.

When interpreting the findings of this investigation, some limitations should be acknowledged. First, our data were collected from one professional team, which was considered a small, but exclusive sample size. Second, the MDS and the distribution of match physical activities relative to the peak demands were only examined using 30- and 60-s epochs. Since greater time epochs are also possible during match-play (Salazar and Castellano Paulis, 2020; Vázquez-Guerrero et al., 2020), using 120-, 180-, and 300-s epochs might be useful to better understand competition. Finally, the exclusive analysis of four variables for physical demands during competition impede to consider contextual factors, such as the activities completed by opponents and team-mates, tactical strategies, and score-line margins. Future research should also consider the distribution of preparatory activities relative to the MDS of match-play during different training sessions (one, two, three, and four days prior to competition) to optimize players’ and team performance.
